# Segmentation of Preretinal Space in Optical Coherence Tomography Images Using Deep Neural Networks

**DOI:** 10.3390/s21227521

**Published:** 2021-11-12

**Authors:** Agnieszka Stankiewicz, Tomasz Marciniak, Adam Dabrowski, Marcin Stopa, Elzbieta Marciniak, Boguslaw Obara

**Affiliations:** 1 Division of Electronic Systems and Signal Processing, Institute of Automatic Control and Robotics, Poznan University of Technology, 60-965 Poznan, Poland; agnieszka.stankiewicz@put.poznan.pl (A.S.); adam.dabrowski@put.poznan.pl (A.D.); 2 Department of Ophthalmology, Chair of Ophthalmology and Optometry, Heliodor Swiecicki University Hospital, Poznan University of Medical Sciences, 60-780 Poznan, Poland; stopa@ump.edu.pl (M.S.); emarciniak@ump.edu.pl (E.M.); 3 School of Computing, Newcastle University, Newcastle upon Tyne NE4 5TG, UK; boguslaw.obara@newcastle.ac.uk; 4 Biosciences Institute, Newcastle University, Newcastle upon Tyne NE2 4HH, UK

**Keywords:** human eye image analysis, preretinal space, retinal layer segmentation, convolutional neural networks, UNet, optical coherence tomography

## Abstract

This paper proposes an efficient segmentation of the preretinal area between the inner limiting membrane (ILM) and posterior cortical vitreous (PCV) of the human eye in an image obtained with the use of optical coherence tomography (OCT). The research was carried out using a database of three-dimensional OCT imaging scans obtained with the Optovue RTVue XR Avanti device. Various types of neural networks (UNet, Attention UNet, ReLayNet, LFUNet) were tested for semantic segmentation, their effectiveness was assessed using the Dice coefficient and compared to the graph theory techniques. Improvement in segmentation efficiency was achieved through the use of relative distance maps. We also show that selecting a larger kernel size for convolutional layers can improve segmentation quality depending on the neural network model. In the case of PVC, we obtain the effectiveness reaching up to 96.35%. The proposed solution can be widely used to diagnose vitreomacular traction changes, which is not yet available in scientific or commercial OCT imaging solutions.

## 1. Introduction

The preretinal space of the human eye and the pathologies connected with its improper changes have become of interest in recent years [1]. The field of ophthalmology has benefited greatly due to the development of noninvasive diagnostic tools such as optical coherence tomography (OCT) [2,3]. This imaging modality uses near-infrared light reflected from the analyzed tissue to illustrate changes in the eye morphology.

As one of the significant advantages, the OCT allows to visualize the progression of posterior vitreous detachment (PVD) and to monitor its possible pathological outcomes [4,5,6,7]. In the majority of the cases, the process of PVD is asymptomatic [8]. It is a consequence of a naturally occurring liquefaction of the vitreous in the aging eye. This phenomenon leads to a progressive separation of posterior cortical vitreous (PCV) [9] from the retina surface, starting from the weakest points of adhesion—the perifoveal quadrants. In the final stages of the detachment, the vitreous separates from the fovea and the optic nerve head.

**Figure 1:**
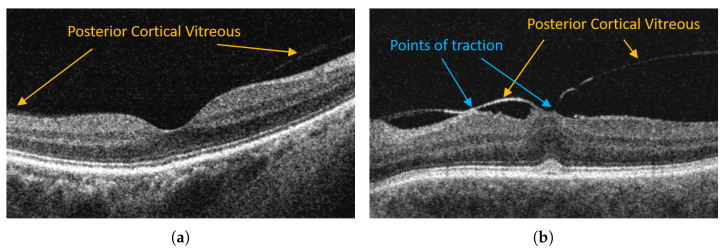
Examples of OCT retina images with posterior vitreous detachment in VMA and VMT. (**a**) Vitreomacular adhesion (VMA). (**b**) Vitreomacular traction (VMT).

Although the complete posterior detachment is prevalent in over 40% of healthy subjects at the age of 60 [8], its abnormal development can cause severe pathological changes such as vitreomacular traction (VMT), epiretinal membrane, and macular hole [10]. For example, Figure 1 illustrates OCT images of a healthy retina with a visible attachment of PCV and a pathological case of vitreomacular traction.

In VMT, the posterior vitreous with continuous adhesion to the macula exerts traction on the retina (mainly fovea—the most sensitive part of the macula). Persistent traction can lead to deformation of the fovea, cystoid foveal thickening, and disorganization of retinal layers. Such problems manifest with metamorphopsia (image deformity), deterioration of visual acuity, blurred or impaired central vision that significantly impair daily tasks (e.g., reading) [11].

It has also been reported that the prevalence of VMT increases significantly with age, from 1% in subjects of 63–74 years old to 5.6% in patients over 85 years old [12]. The chance of spontaneous VMT resolution is up to 23% [13]. However, if left untreated, the probability of severe retina damage (frequently due to the development of a macular hole) and vision deterioration increases with time. Systematic monitoring of preretinal changes allows the physician to determine if (and when) surgical intervention is required.

Currently, VMT advancement is determined based only on a single cross-section through the center of the macula. To the best of our knowledge, no research or commercial image informatics solutions allow for automatic segmentation of the vitreous cortex and thus the preretinal space. Therefore, manual measurements can only be made in a few points of the volume and are not sufficient to quantify the profile of this epiretinal pathology. A numerical analysis of preretinal space volume and adhesion area is required to assess precisely the development and current stage of VMT. Such investigation can include: quantification of the preretinal space volume [14], statistical analysis of vitreoretinal interface parameters (e.g., adhesion area), description of the stage of pathology development [15] and its changes in time. Therefore, the eye doctor community highly desires fully automated OCT image analysis presented in this paper as a necessary step in advancing the fundamental understanding of the changes in vitreoretinal interface pathologies. Automated segmentation of preretinal space and thus 3D volumetric analysis would optimize available treatment strategies, including ocriplasmin, sulfur hexafluoride, and octafluoropropane gas injection [14].

### Key Contributions

The focus of this paper is the development of an automatic image informatics approach for the segmentation of the preretinal space in both healthy and pathological cases. The major contributions of this study are:

- Evaluation of state-of-the-art neural network methods employed for the new task of preretinal space segmentation to the best of our knowledge has not been previously attempted. The conducted experiments demonstrate that the deep learning approach is better suited for segmenting preretinal space than a standard graph-search method.
- Analysis of the influence of kernel shape and size on convolutional network performance for OCT images. With the experiments described in Section 6, we show that by changing the shape and size of a convolutional kernel, it is possible to overcome topology errors in pixel-wise retina layer segmentation.
- Analysis of relative distance map calculation to improve retina layers topology preservation using various network architectures. We propose two methods of obtaining a distance map for any given image. They do not require any given knowledge about the retina layers (as is the case in the compared methods), are less computationally expensive, and give similar (or in some cases better) results.
- Collection of a unique dataset of 3D OCT images from 50 VMA and VMT subjects (7050 2D B-scans) with manual segmentation data of preretinal and retinal volumes [16]. The gathered data was statistically analyzed with respect to image quality and features distribution.

The rest of the article is organized as follows. First, the recent state-of-the-art approaches for retina layers segmentation as well as the methods selected for preretinal space segmentation are described in Section 2. Section 3 contains the characterization of the data used in the study. Section 4 presents the methods implemented in this study, while Section 5 describes the experiment setup. The results of the designed experiments are presented in Section 6. Finally, Section 7 summarizes the conducted research, discusses its advantages and limitation, and provides insights into problems worth investigating in future studies.

## 2. Related Works

### 2.1. Retinal Layers Segmentation

With the increased availability of OCT imaging devices, the detailed analysis of retina pathologies became possible. However, manual segmentation and measurement of tissue biomarkers are very time-consuming, and with an increased number of pathological subjects, not always an option. Thus, multiple image informatics algorithms have been developed in recent years to support the effort of ophthalmologists.

Development of the algorithms for retina layers segmentation started over 20 years ago with classical image informatics approaches, that can be divided into: edge detection methods [17,18,19,20], active contour modeling [21,22,23], and graph search and dynamic programming [24,25,26].

As can be derived from analysis of the literature, the complexity of proposed methods advanced with years, as well as their accuracy of retina layers segmentation and number of layers that could be segmented. Since 2012 the graph-search methods proved one of the most accurate retina layers segmentation for healthy and pathological cases. Their disadvantage, however, is the need for extensive image preprocessing (primarily noise suppression) [27,28] and careful selection of parameters for each dataset to make the designed approach suitable for the task. Additionally, the complexity and high time consumption make them inadequate for real-time application in a clinical setting.

The progress has also been made with machine learning, pattern recognition, kernel, and clustering techniques [29,30,31]. Furthermore, after the expansion of convolutional neural networks (CNNs) in the field of image segmentation, fully convolutional networks (FCNs) became a useful tool for segmentation of biomedical images [32,33,34]. In 2017, the first attempt was made to use a network called ReLayNet [35] (a variation of UNet [33] and DeconvNet [36]) for retina layers segmentation.

Table 1 lists a summary of neural network topologies utilized in current pixel-wise approaches for retina layers segmentation (excluding papers that combine pixel-classification with graph search technique). In addition, the literature was analyzed in terms of the number and size of images utilized for training and experiment settings (e.g., segmented layers, loss function, data augmentation methods). Any previously undefined diseases abbreviations are described at the end of the paper.

The reviewed works focus on segmenting main retina layers in normal and pathological subjects and, in some cases, accompanied fluids. Although the available databases consist of a limited number of training images, researchers compensate for that with data augmentation methods. The majority of published methods are based on UNet architecture [33], its modifications (with dilated convolutions [40], batch normalization [35], dropout layers [42]) or combination with other networks, such as ResNet [45]. Aside from accuracy, the advantage of the facilitation of neural networks for retina segmentation is their ability to simultaneously segment multiple layers as well as fluids and pathological tissues. Furthermore, contrary to classical solutions, they do not require a separate set of models and parameters for each specific disease or normal case.

### 2.2. Preretinal Space Segmentation

Despite the plethora of available retina layer segmentation methods, preretinal space segmentation from OCT images is not widely researched. To the best of our knowledge, deep learning methods have not yet been used for this task, and only a handful of reports of other approaches can be found in the literature.

For instance, Malagola et al. [46] showed that it might be possible to measure the volume of the preretinal space after re-calibrating the native OCT device segmentation algorithm to search for the preretinal space instead of the retina. This approach, however, does not allow to perform any further numerical analysis concerning the retina morphology, is not fully automatic, requires re-calibration of the device for each scan, and most of all, is not device-independent, thus cannot be employed in worldwide research.

The first graph search-based approach that segmented both the retina borders (e.g., inner limiting membrane (ILM), and retinal pigment epithelium (RPE)) and the posterior vitreous cortex was published in 2014 [47]. However, as was reported, the graph search requires significant preprocessing (e.g., denoising, removing low quality areas of the image). Even with such preparations, this method is prone to errors due to the fact that the PCV line is frequently on the level of noise in the image or its reflectivity is too low. The main disadvantage of the graph search approach is the assumption that the PCV line is visible through the entire width of the image, which, due to its varying density, is not necessarily true.

Furthermore, automated segmentation was used for quantitative analysis of epiretinal abnormalities only in several studies [48,49,50] (for epiretinal membrane and macular hole). The lack of research in PCV segmentation is caused by the unavailability of the data (OCT scans and manual segmentation) with both VMA and VMT, and insufficient accuracy of state-of-the-art retina segmentation methods when applied to this task.

### 2.3. Issues with Layers Topology

As was described above, the convolution neural networks typically output probability maps classifying each pixel belonging to one of the designed classes. However, it means that individual pixels are analyzed locally, while this methodology gives results that may perfectly match regional data, if we look closely at a B-scan (see Figure 1), it can be noticed that many areas belonging to different layers or image regions have similar intensities or contrast characteristics. As a result, CNNs produce inconsistencies in the topological order of retina layers that are unacceptable in medical imaging [51].

One of the first approaches to address that issue was proposed by He et al. [44,51]. In their works, two separate networks were used: first to learn the intensity features and second to correct the obtained topology by learning the implicit latent network features corresponding to shape factors in an adversarial manner. Some other works on topology-guaranteed predictions tried to directly predict the coordinates of layer boundary while encoding prediction of lower layers as a relative position to the upper ones [52]. Nevertheless, such an approach may easily lead to error propagation if the uppermost boundary is incorrectly segmented.

A promising approach was proposed by Lu et al. [53], in which they integrated information about the hierarchical structure of the image in the form of a relative distance map (RDM). This map, computed from an initial graph search-based retina borders segmentation in a preprocessing step, was provided to the neural network as a second channel of the input image. This work was further extended by Ma et al. [40] by substituting the graph search-based initial segmentation with cascading networks trained separately.

The relative distance map provides a way of introducing additional spatial information alongside the input image. Each pixel value of the map corresponds to the pixel position in the image as a function of retina location. Thus, knowing the coordinates of inner and outer retina borders (namely ILM and RPE) across a B-scan, the intensity values of the relative distance map are computed for each pixel with indexes (x,y) as follows:

(1) $$M\left(x,y\right)=\frac{y-ILM(x)}{RPE(x)-ILM(x)}\;,$$

where ILM(x) and RPE(x) represent the *y* (vertical) coordinate of previously segmented ILM and RPE lines in the image column *x*. According to this equation, the pixels above the ILM take value <0, pixels positioned within the retina tissue take values in the range of 〈0,1〉, and pixels below the retina are >1. Such weighing scheme, concatenated (as a second channel) to an original OCT image, which is also in the range of 〈0,1〉, allows the network to learn layers topological dependence. Such procedure boost precision of segmenting non-neighbouring layers with similar intensity patterns or lower contrast.

Our work explores the possibility of utilizing deep neural networks (DNN) for the task of preretinal space segmentation and investigates the challenges connected with the specificity of the preretinal space (shape and image intensity variations). We present the research results for preserving the topological correctness of the segmented OCT image areas, including the implementation of four different distance maps (based on prior segmentation or without it). We propose investigating the influence of convolutional kernel size and shape on topology correctness and overall segmentation accuracy.

## 3. Materials

### 3.1. OCT Image Dataset

The goal of conducting research in OCT retina layers segmentation creates a need to obtain numerous images with annotated biomarkers searched for in a specific task. Since 2018 only several public OCT databases with 3D images have been established, and most of them are aimed at classifying a specific disease from a single B-scan. Other cohorts, focused on automatic retina layer segmentation, provide manual segmentations for 3 to 8 retina borders. Most contain images of 10 to 25 volumes [37,54,55,56], although one database consists of over 380 scans [57]. The subjects included in those databases are either healthy or involve patients suffering from pathologies such as age-related macular degeneration (AMD), diabetic macular edema (DME), diabetic retinopathy (DR). Nevertheless, none of the available databases concern patients with vitreoretinal interface pathologies, especially vitreomacular traction, not to mention annotations of PCV.

Thus, a CAVRI (Computer Analysis of VitreoRetinal Interface) dataset [16] of OCT images with VMA and VMT has been created to analyze the characteristics of preretinal space. Subjects for this dataset were recruited at the Department of Ophthalmology, Chair of Ophthalmology and Optometry, Heliodor Swiecicki University Hospital, Poznan University of Medical Sciences in Poznan, Poland. The study was approved by the Bioethics Committee of Poznan University of Medical Sciences under resolution no. 422/14. All participants signed an informed consent document before enrollment.

The CAVRI database contains 3D images of the macula obtained with the Avanti RTvue device (Optovue, Incorporated, Fremont, CA, USA) using the 3D Retina scanning protocol. For this research, from a group of 73 cases a set of 50 OCT volumes was selected: 25 examples of the healthy retina (with asymptomatic vitreomacular adhesion (VMA)) and 25 subjects with VMT. Each 3D scan consists of 141 cross-sections with 640×385 px resolution representing 2×7×7 mm retina volume. The corresponding voxel sizes equal 3.125 μm in the vertical direction (further denoted as *y*), and 18.18 μm and 49.65 μm in fast-scanning (*x*) and non-fast scanning (*z*) directions, respectively. No multi-sampling and noise-reduction protocols were used during acquisition or preprocessing. All 7050 cross-sections (also called B-scans), visualized as gray-scale images, were analyzed separately.

The PCV line, visible as a hyperreflective line in a B-scan (see Figure 1), was manually annotated under the supervision of clinical experts (three ophthalmologists) using a custom-made public software OCTAnnotate [58]. Selected 50 subjects had a maximum difference of manual segmentation between the experts less than 3 px. In addition, two other lines denoting retina borders were also labeled for investigational purposes, namely the inner limiting membrane (ILM) and retina pigment epithelium (RPE) outer border. These three lines (and consequently four image regions they create) are the ground truth for this research. Based on reference segmentation of PCV and ILM lines, we calculated the preretinal space volumes as the main metric of comparison. The average value of preretinal space volume for VMA is 3.17±1.96 mm${}^{3}$ and for VMT is 12.19±6.09 mm${}^{3}$. The Wicoxon-test resulted in *p*-value close to zero ($3.6\times 10^{-10}$), which confirms significantly different preretinal space volumes for the VMA and VMT groups.

### 3.2. Data Anomaly Detection

As part of a data-driven field of artificial intelligence, deep neural networks are highly dependent on the data itself. As is widely considered, the more data used for the training, the better the machine learning model [59]. Additionally, when providing data for the training, one should ensure that all classes (data categories) are equally represented in the dataset to give the model a chance to learn characteristic features of all cases possible to occur in the evaluating set. Furthermore, the quality of the obtained model increases with better data samples [60]. Accordingly, anomalous examples may hinder the learning process regardless of the complexity of the model.

By performing statistical analysis of the data, it is possible to discern the anomalous examples in the set. The outliers (otherwise known as anomalies or exceptions) are data examples outside the normal distribution of data features [61,62]. For images, it is possible to distinguish multiple descriptive features (from size and color intensity to contrast and noise). Outliers then could be found in an n-dimensional space (of n-features) [63].

As was established in [64] the presence of outliers in the training dataset could significantly compromise the accuracy of a machine learning model. Therefore, detecting and removing data samples that in any way differ from the bulk of the training set have a favorable influence on obtaining a better and more robust prediction model [65,66].

A wide variety of outlier detection algorithms are constantly developed and compared by many researchers [67,68,69]. In our experiments, a robust covariance method [70] was utilized for this task. The advantage of this unsupervised anomaly detection method is a fast estimation of unusual data without the need for labeling or any prior knowledge about the dataset. This technique calculates the elliptic envelope of the features (assuming a Gaussian distribution of the entire dataset) and regularizes the covariance matrix to determine the samples outside the boundary. The m% of the images with the lowest prediction scores are considered anomalous.

**Figure 2:**
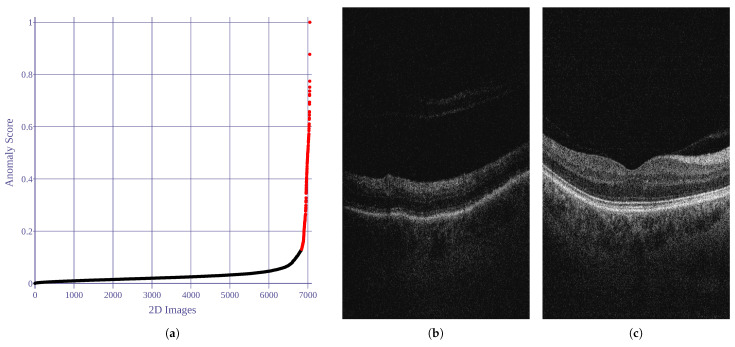
Results of 2D image anomaly detection. Anomaly score is computed as 1− robust covariance value. Black and red dots represent normal and abnormal images, respectively. (**a**) Anomaly scores for all images. (**b**) Anomalous example (score = 1). (**c**) Normal example (score = 0).

Utilizing the anomaly detection implementation provided by Kucukgoz [71], five image features are employed in our research, namely: noise score, contrast score, brightness-darkness score, blurriness score, and average pixel width score. Based on the estimated covariance predictions, 3% of the data samples with the lowest score were established as anomalies and excluded from the experiment. Figure 2 illustrates the anomaly scores (=1− covariance score) for the analyzed images.

## 4. Methods

To segment the PCV using a deep learning approach, we trained four state-of-the-art convolutional neural networks. Then, we compared their output with the previously described graph search-based approach and the ground truth. This section gives a general description of the utilized fully convolutional network architectures, including UNet, Attention UNet, ReLayNet, and LFUNet.

**Figure 3:**
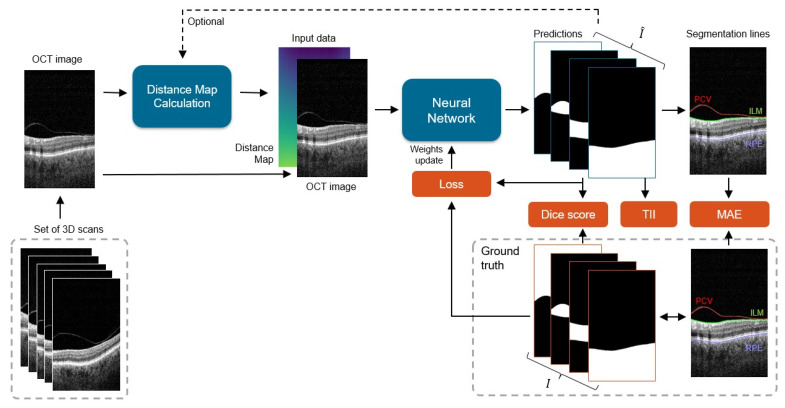
General scheme of the processing pipeline: each 2D slice from a set of 3D scans is processed by the network to obtain four prediction masks (one for each segmentation class), from which 3 area borders are calculated: posterior cortical vitreous (PCV), inner limiting membrane (ILM), and retina pigment epithelium (RPE). Predicted segmentations $\hat{I}$ are compared with the ground truth data *I*, and the difference between them (in the form of loss) is used to update the network weights. During the test phase, error metrics of Dice score, Mean Absolute Error (MAE), and Topology Incorrectness Index (TII) are computed.

The processing pipeline of the proposed system is presented in Figure 3. Our framework learns correct preretinal space segmentation by separately processing a cohort of 2D OCT cross-sections with their relative distance maps. The predicted probability maps are compared with the ground truth, and the resulting error (loss) is used to update the network weights. The final binary segmentation maps are used to calculate borders between the segmented image regions, namely the PCV, ILM, and RPE lines.

### 4.1. Preretinal Space Segmentation

#### 4.1.1. Graph Search Approach

The classical image segmentation utilizing graph search and dynamic programming is described in [26]. Here, based on the vertical image gradient, an adjacency matrix for a graph is calculated. Next, the algorithm discerns the shortest path between the left and the right border of the image. This path represents a cut between image regions such as preretinal space and retina (i.e., ILM line) or retina and choroid (i.e., RPE line).

As was described in [47], the same principle can be applied to segmenting the edge of the posterior vitreous cortex. The areas between the subsequent lines correspond to preretinal and retinal regions.

#### 4.1.2. UNet

UNet is an architecture proposed by [33] that obtains good accuracy in semantic segmentation of biomedical images. It consists of encoder and decoder paths, each with five levels of two convolution blocks. Each block incorporates a 3×3 px convolution followed by ReLU (Rectified Linear Unit) activation function. Between each of the five encoding level, a downsampling 2×2 px max-pool operation with a stride 2×2 px is applied. Simultaneously, each level doubles the number of feature channels. Consequently, the feature maps are upsampled with a 2×2 px up-convolution in the decoder path while halving the number of feature channels.

One of the beneficial procedures introduced in UNet is a skip connection: e.g., the feature maps at the end of each encoder level are concatenated to the upsampled decoder maps before being processed by the convolution blocks. Such operation allows preserving relevant information from the input features. The probability maps are obtained after applying a final 1×1 px convolution after the last decoder block, transforming the 64 element feature matrix into a segmentation mask for each desired class.

#### 4.1.3. Attention UNet

An extension of the UNet architecture is the Attention UNet proposed by [72]. It introduces attention gates to highlight any significant features that are passed through the skip connection. Its advantage is maintaining a simple design while decreasing model sensitivity to the background regions.

The general design of this network is similar to the baseline UNet, with five double 3×3 px convolution blocks in the encoder and decoder paths. The attention module is applied to each encoding result before they are concatenated to the decoder blocks. The function of this grid-based gating mechanism is to minimize the influence of irrelevant or noisy features. The PyTorch implementation of the Attention UNet network utilized in this experiment was obtained from [73].

#### 4.1.4. ReLayNet

ReLayNet [35] was the first CNN employed for the retina layer segmentation task. It is based on UNet, but with fewer convolution layers in each encoder and decoder block, a non-expanding number of features in each hidden layer, and only 3 (instead of 4) pooling/unpooling operations. An addition to such simplified architecture is the Batch Normalization procedure performed after each convolution and before the ReLU activation function.

The ReLayNet also differs from the original UNet with the kernel size used for each convolution, which is 7×3 px instead of 3×3 px. As was reported in [35], this ensures that the receptive field at the lowest level in the network covers the entire retina depth. As will be further proved in Section 6, increasing the receptive field of a convolution kernel has a significant impact on the segmentation accuracy.

#### 4.1.5. LFUNet

The LFUNet network architecture is a combination of UNet [35], and FCN [74] with additional dilated convolutions [75]. In this network, the encoder part is the same as in the original UNet and consists of 4 blocks that contain two convolution layers with kernel size 3×3, and a 2×2 px max pooling layer with stride 2.

The decoder part consists of two parallel paths for UNet and FCN. The UNet path utilizes concatenation of up-sampled feature blocks with the corresponding blocks from the encoder part (a procedure also referred to as “skip-connections”, that allows exploiting high-resolution information). The FCN path performs the addition of up-sampled feature blocks with the matching encoder blocks. The upsampling in both paths is performed with the 2×2 px up-convolution layer after each convolution block.

The additional strength of this network introduces the last part, which is a concatenation of final feature maps obtained from both decoder paths. They are subsequently dilated with three separate kernels, and the resulting matrices are again concatenated before final convolution. The output probability map for each pixel belonging to one of the *C* classes was obtained with the Softmax function. ReLu was used as all activation functions in the hidden layers.

### 4.2. Relative Distance Map

It should be noted that the problem of preserving correct topology in retina layers segmentation is even more pronounced for the preretinal space since it has almost the same intensity range as the vitreous. Hence, in this work, we employed the available approach for preparing the RDM (here referred to as “2NetR”) based on prior information of retina borders and proposed a modified version tailored to the problem of segmenting the preretinal space. In addition, we also tested if a more straightforward map that does not require two cascaded networks and is computationally less expensive could also facilitate this task.

#### 4.2.1. RDM Based on Prior Segmentation

To increase the significance of preretinal space as a region below the vitreous but above the retina, we propose to utilize a distance map (further called “2NetPR”), that would take the following values:

- For vitreous region: M(x,y)∈(−∞,0);
- For preretinal space: M(x,y)∈〈0,0.5);
- For retina: M(x,y)∈〈0.5,1〉;
- For region below retina: M(x,y)∈(1,∞).

This can be defined for each pixel with the following formulations:

(2) $$M\left(x,y\right)=\left\{\begin{array}{cc} \frac{y-PCV(x)}{RPE(x)-PCV(x)} & \mathrm{if}\;y<PCV(x)\;\mathrm{or}\;y>RPE(x),\\ \frac{1}{2}\frac{y-PCV(x)}{ILM(x)-PCV(x)} & \mathrm{if}\;y\in \langle PCV(x),ILM(x)),\\ \frac{1}{2}\left(\frac{y-ILM(x)}{RPE(x)-ILM(x)}+1\right) & \mathrm{if}\;y\in \langle ILM(x),RPE(x)\rangle . \end{array}\right.$$

Nevertheless, as efficient an idea as this is, it still requires prior knowledge of retina borders in a given cross-section. As reported, this information can be obtained via graph search approach [53], or by performing the segmentation twice, incorporating two neural networks [40].

#### 4.2.2. RDM without Prior Segmentation

Thus, we also investigated an approach that does not require any a priori knowledge about the retina position within the analyzed image. Two following solutions are evaluated:

- Basic Map with Orientation—Firstly, we investigated if a map of linearly spaced values in the range of 〈0,1〉 would provide the network with sufficient information about the layers’ hierarchy. Additionally, to account for retina orientation in the image and resulting rotation of the preretinal space, we propose to arrange the values according to the said retina orientation. For this purpose, the orientation is determined by first applying a Gaussian filter on the image (with σ=3) and then calculating *arctan* of the vertical and horizontal image edges subsequently obtained with the use of Sobel edge detection. This map will be further called “BasicOrient”.
- Cumulative Sum Map—The second method incorporates calculating a cumulative sum of intensity image values for each column of the image. This is based on the assumption that pixels in the vitreous and preretinal space region have very low-intensity values, as opposed to the retinal region. Additionally, the pixels below the retina have average intensity, hence providing lower variations in the cumulative sum. Furthermore, by performing a scaling operation, it is possible to obtain values similar to those produced by Equation (1), but with the significantly less computational expense (no need to use initial segmentation). Furthermore, this method is not subjected to error propagation (which may occur if the initial segmentation algorithm provides incorrect ILM and RPE borders). Thus, this map is further referred to as the “CumSum” map.

**Figure 4:**
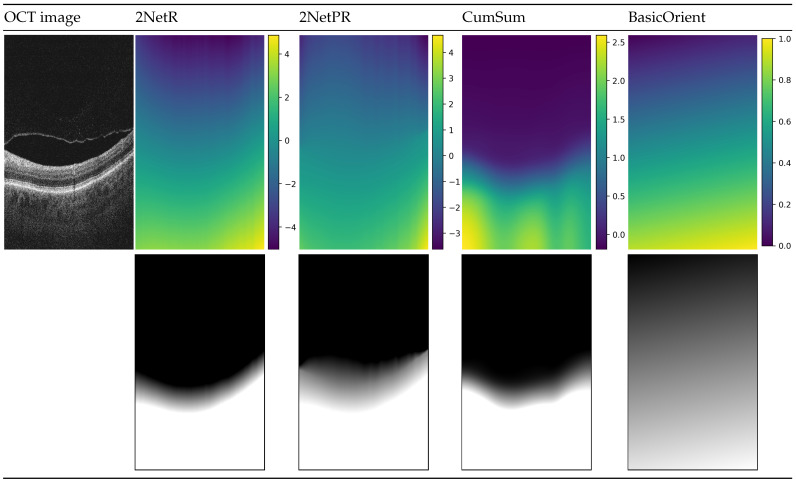
Visualization of used distance maps for an example B-scan: “2NetR”, a map based on previous retina segmentation with a neural network, “2NetPR”, a map based on previous segmentation of retina and preretinal space, “CumSum”, a Cumulative Sum Map, and “BasicOrient”, a Basic Map with Orientation.

Figure 4 illustrates prepared distance maps, for an example input OCT image, using four methods described above. The color in the images in the first row depicts values in the range given for each distance map. The images below represent the same map in greyscale, for which values are in the range of intensity of a B-scan image (i.e., values below 0 are represented by a black color, values in the range of 〈0,1〉 are in the shade of gray, values bigger than 1 are white).

### 4.3. Kernel Size

The pixel intensity values of the preretinal space are similar to those of the vitreous. Therefore, the network may not have enough information about the surroundings to correctly assign a given pixel to a class. Furthermore, the area and shape of the preretinal space differ from B-scan to B-scan.

Another way of providing the network with the information of where a given pixel belongs within the image is using a bigger convolution kernel. In contrast to works described in the literature review in Section 2, we propose the use of a non-typical convolutional kernel. It has been reported that by utilizing a vertical convolutional kernel of the size 7×3 px for ReLayNet [35], the network captures the entire retina in the lowest convolution level. Nevertheless, this approach has not been sufficiently discussed or analyzed in retina layers segmentation to explain the selected kernel size.

Within the retina scan pixel intensities vary significantly in the vertical direction, therefore it can be beneficial to utilize a bigger kernel to detect those changes. In our experiments we check the influence of:

- square kernels: 3×3, 5×5, and 7×7 px,
- vertical kernels: 5×3, 7×3, and 9×3 px,
- horizontal kernels: 3×5, 3×7, and 3×9 px.

Bearing in mind the computational cost of incorporating a bigger convolutional kernel, we pose that even a non-uniform filter will significantly improve the accuracy of pixel-wise segmentation.

## 5. Experiment Design

We have performed a comprehensive set of experiments designed to measure the performance of various deep neural networks employed for the segmentation of preretinal space from OCT images. We evaluated the effect of removing anomalous data samples from the training set and the influence of data augmentation on the model accuracy. We addressed the issue of incorrect class topology common in pixel-wise image segmentation with the calculation of a relative distance map as guidance information for the system. With a set of tests we compared our proposed solution to a state-of-the-art method. Furthermore, we also proposed and evaluated an alternative method of changing the size of the convolution kernel while measuring the computational complexity.

In this section, we describe the experiment setup and parameters of the system for all segmentation methods. Next, we describe data augmentation techniques utilized in this research, and finally, we provide information regarding the evaluation metrics used to compare the obtained results quantitatively.

### 5.1. Training

The goal of the segmentation task is to predict 4 separate areas in the image, further described as set of classes C={0:Vitreous,1:PreretinalSpace,2:Retina,3:Spaceunder Retina}. The training network’s task of multi-class classification is to assign each pixel of the image to one of these classes.

Bearing in mind the specificity of preretinal space, we consider the possibility that the PCV line is not sufficiently visible throughout the scan or is partially connected to the ILM. In such a situation, using narrow patches could mislead the network. Hence, we input to the network an entire B-scan (e.g., gray-scale image) with the resolution of 640×384 px, what encourages a smoother layer surface across the image. In addition, each image before processing was subjected to a standard *z*-score normalization.

All neural networks described in this paper were implemented using Python 3.7 with PyTorch 1.8.1 and NVIDIA CUDA 11.2 libraries. The experiments were conducted on a 64-bit Ubuntu operating system with an Intel Core i7-7700K 4.20GHz computing processor and 32 GB RAM. The NVIDIA GeForce GTX 1080 Ti GPU card with 11 GB memory was used during training and evaluation.

The CAVRI dataset was randomly split into training, validation, and testing subsets with the ratio of 80%, 10%, and 10%, respectively. The images in the training set were used to learn the neural network weights. The validation set was used at the end of each epoch to check the model’s accuracy and validation loss. Finally, the test set contains images previously unseen by the network and is used to evaluate all segmentation methods.

Using the PyTorch Lightning 1.3.5 library, we trained each network with an Adam optimizer and the following parameters: learning rate $l_{r}=5\cdot 10^{-6}$, $\beta_{1}=0.9$, $\beta_{2}=0.999$. Due to the random cropping procedure used for data augmentation, which produces images of various sizes, the batch size was set to one. Each network was trained for at least 50 epochs, and the training was stopped if the validation loss did not decay for the last five epochs. Models were evaluated on the best checkpoint corresponding to the lowest validation loss value. It should also be noted that due to memory constraints, all networks were implemented with 32 initial feature vectors instead of the original 64. According to the initial experiments, this change, however, does not have a significant degrading impact on model accuracy. The hyper-parameters of the model (i.e., weights of the loss function, data augmentation techniques) were chosen experimentally, and the best values and techniques were used to obtain the presented results. The implementation code is available online at https://github.com/krzyk87/pcv_segmentation, (accessed on 5 November 2021).

The graph search algorithm was implemented in the Matlab/Simulink environment [76] on a 64-bit PC workstation with Windows 10 operating system, Intel Core i7-3770 3.40 GHz processor, and 8 GB RAM.

#### 5.1.1. Loss Function

The training is aimed at minimizing a loss function $L(I,\hat{I})$ between the arrays of ground truth *I* and prediction $\hat{I}$. It is designed as a weighted sum of multi-class logistic loss ($L_{log}$) and Dice loss ($L_{Dice}$). The utilized loss function is implemented as it was proposed in the referenced and compared methods [35,40] to sustain a consistency between each network architecture. The multi-class log loss, otherwise known also as Categorical Cross-Entropy, is a type of distribution-based criterion calculated as follows:

(3) $$L_{log}\left(I,\hat{I}\right)=-\sum_{c\in C}\left(\frac{1}{n_{c}}\sum_{x,y}\omega_{c}\left(x,y\right)\cdot I_{c}\left(x,y\right)\cdot \log\left({\hat{I}}_{c}\left(x,y\right)\right)\right),$$

where $I_{c}\left(x,y\right)$ is a binary ground truth mask for class c∈C={0,1,2,3} taking value 0 or 1 at each location (x,y), for x∈X={1,…,w} and y∈Y={1,…,h}, where *w* and *h* denote the width and height of the image, respectively; ${\hat{I}}_{c}\left(x,y\right)$ is the prediction probability of the pixel with indices *x* and *y* belonging to class *c*; $n_{c}$ is the number of pixels in a given class *c*; and $\omega_{c}\left(x,y\right)$ is an additional weight given to each pixel depending on its class and position within it.

In detail, since the PCV line is very often on the level of noise in the image and due to OCT characteristics, the edges of the regions can be blurred. To boost the network’s sensitivity to class boundaries, the pixels at the edges are given an additional weight $q_{1}$. Furthermore, the pixels belonging to classes of interest (namely preretinal space and retina) are given an additional weight $q_{2}$ to adjust for their lower area in the image (as opposed to the background). Equation (4) describes the overall pixel weight calculation:

(4) $$\omega_{c}\left(x,y\right)=1+q_{1}\cdot f(|\nabla_{y}I_{c}\left(x,y\right)\left|>0\right)+q_{2}\cdot f\left(I_{c}{\left(x,y\right)}_{|c=1,2}\right),$$

where f(*) is an indicator function taking a value of one if the (*) is true, and else zero. The $\nabla_{y}$ operator represents the vertical gradient.

The second component of the loss function is computed based on a Dice coefficient. The Dice as region-based metric measures the overlap of two regions according to the equation:

(5) $$Dice_{c}=\frac{2|I_{c}\cap {\hat{I}}_{c}|}{|I_{c}\left|+\right|{\hat{I}}_{c}|},$$

where |*| denotes a sum of pixels in the corresponding mask of ground-truth $I_{c}$ and prediction ${\hat{I}}_{c}$ for a class *c*.

Consequently, the Dice loss $L_{Dice}$ takes into account the Dice scores for all the classes and can be expressed as follows:

(6) $$L_{Dice}\left(I,\hat{I}\right)=1-\sum_{c\in C}\lambda_{c}Dice_{c}\left(I_{c},\hat{I_{c}}\right),$$

where $\lambda_{c}$ is a weight assigned to each class to compensate for their imbalance within the set. Numeric analysis of all the pixels in the dataset belonging to each class shows that the preretinal space is the most underrepresented class, while the background (vitreous region even more than the region below the retina) spans the largest area in each volume. We calculated the weights for each class as presented in Table 2 using the following equation:

(7) $$\lambda_{c}=\frac{\frac{1}{n_{c}}}{\sum_{c}\frac{1}{n_{c}}},$$

where $n_{c}$ is the number of pixels belonging to the class c∈C. All the weights sum up to 1, so that a maximum Dice score for all the classes would produce a Dice loss equal to 0, according to Equation (6).

The overall loss function $L(I,\hat{I})$, being a weighted sum of the above-described formulas, is calculated as follows:

(8) $$L\left(I,\hat{I}\right)=\alpha L_{log}\left(I,\hat{I}\right)+\beta L_{Dice}\left(I,\hat{I}\right),$$

where α and β are the weights assigned to each loss component. During the experiment, their values were empirically chosen as α=1 and β=0.5. The parameters for pixel-wise weight in Equation (4) are also consistent with the compared methods: $q_{1}=10$ and $q_{2}=5$.

#### 5.1.2. Data Augmentation

**Figure 5:**
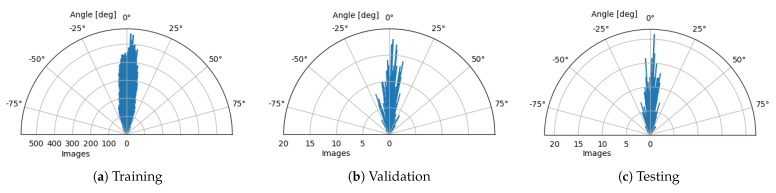
Circular distributions of retina orientations in each subset of the CAVRI dataset.

**Figure 6:**
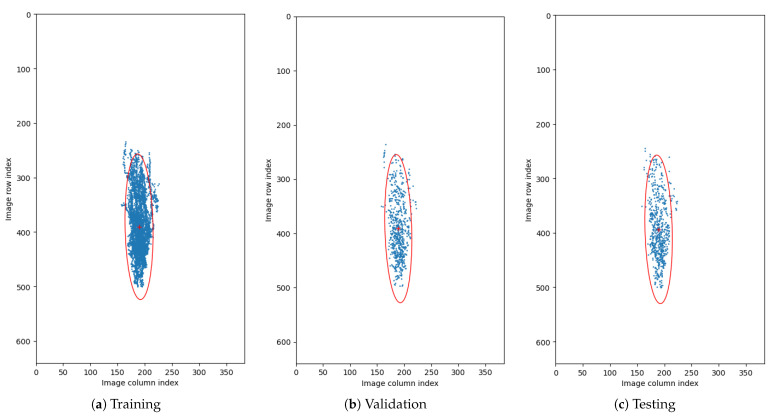
Distribution of center of mass of images. Blue dots represent calculated position of center of mass for each OCT cross-section. Red ’+’ sign denotes the mean center of mass, and the red ellipsis encapsulates the 2.5 standard deviation of the data. The data is presented on the plane with resolution equal to those of the OCT cross-sections.

We utilized data augmentation techniques during training to improve the model’s generalisability and increase the segmentation accuracy. Thanks to this, the number of the image examples expanded artificially with each technique while maintaining the data characteristics that may naturally occur in the data. The following transformations performed in 2D for each cross-section were used:

- Horizontal Flip—Allows obtaining a mirrored image which coincides with having a scan of the other eye (left for right and right for left), preserving the morphology of retinal structures (such as vessels and layers topology).
- Rotation—Slight variations in retina orientation are natural when acquiring an OCT scan. Thus, augmenting images in this way allows training the model to anticipate any future examples in a wide variety of retina orientation angles. In order to determine the range of random orientations to apply, we performed a statistical analysis of a retina orientation distribution within the CAVRI dataset. As can be seen in Figure 5, the obtained results for all subsets have similar distribution and are within ±25 degrees. Thus, a rotation with a randomly chosen angle in the range of ±20 degrees was performed for each image.
- Vertical Translation—Automatic acquisition protocol in an OCT device aims at focusing the device’s optics on the retina. Notably, the thickness of the retina tissues extends to an average of 200 μm within a 2 mm depth of the scan. Therefore, we performed a statistical analysis of the retina position within the image (across all cross-sections in the database) to determine the retina vertical position distribution. For that purpose, we estimated the center of mass in each image and plotted the obtained positions within the image dimensions range as illustrates Figure 6. It can also be noted that each subset maintains a similar distribution, confirming appropriate dissemination of samples between the subsets. Based on the gathered information, we set the range of vertical translation of the image to ±10% of the image height, equal to ±64 px.
- Random Crop—The wide variety of OCT acquisition devices allow for an even greater number of scanning protocols, with various image sizes and scanning widths. Thus, performing an augmentation technique of random cropping, we train the network to perform well on any input image regardless of its size or fovea width to image width ratio. In our experiment, we employed a crop with randomly selected values for both width and height (within the range of 80–100% of the original values).

Utilizing such data augmentation techniques allowed to increase the number of training examples, as shown in Table 3.

### 5.2. Evaluation Metrics

To compare the correctness of the proposed segmentation methods with the manual annotations, we employ the following evaluation metrics:

1. Dice Coefficient—A measure of overlap between the segmented region in the ground truth and prediction. It is calculated for each of the segmented classes (Vitreous, Preretinal Space, Retina, Region under Retina) based on Equation (5) separately and averaged over all test samples. Values closer to 1 represent better performance. The standard deviation (SD) values illustrate scores distribution.
2. Mean Absolute Error (MAE) with standard deviation (SD)—The average vertical distance between an annotated line and a segmented boundary. It represents the error of the segmentation result relative to the ground truth. It is calculated as follows:
  (9) $$MAE_{z,b}=\frac{1}{w}\sum_{x=1}^{w}|P_{z,b}\left(x\right)-G_{z,b}\left(x\right)|,$$
  where P(x) and G(x) are the vertical position of the class boundary line b∈B for the prediction and ground-truth, respectively; *x* stands for the image column index within the image width *w*; and *z* denotes an individual image index. The MAE is computed for the three segmentation lines of interest, namely the B={0:PCV,1:ILM,2:RPE}, and is averaged over the number of all B-scans used in the test.
3. Topology Incorrectness Index (TII)—that indicates what percentage of tested images has incorrect layers topology in the vertical direction. It is computed based on a vertical gradient of the predicted segmentation mask.

## 6. Results

### 6.1. Preretinal Space Segmentation Accuracy

We conducted multiple experiments using several networks to segment the preretinal space and the retina in the OCT images. This section presents a qualitative and quantitative comparison of preretinal space segmentation with a graph search approach and four DNN methods. Specifically, UNet, LFUNet, Attention UNet, and ReLayNet networks were used to obtain precise segmentation masks. Table 4 presents baseline results for a whole dataset, without any additional strategies described in this paper. The Dice and MAE (SD) metrics show how accurate the performed segmentation is.

As can be noticed, all neural networks perform better than the graph search-based method, and from CNN, the UNet has the best performance in all segmented areas and borders. On the other hand, the ReLayNet gives the worst results, which may be explained by a relatively lower number of features compared to other architectures. Additionally, the preretinal space boundary of PCV and the image classes it separates (i.e., Vitreous and Preretinal Space) have worse accuracy than the clearly defined ILM and RPE borders and the two image regions they define. This confirms the difficulty of determining preretinal space boundary due to similar pixel intensities of this region to the vitreous part.

### 6.2. Effect of Removing Anomalous Data Samples

In Table 5, we demonstrate how removing anomalous data samples from the dataset can improve the training of the network. The results show that the accuracy improved for all the tested methods. Here the LFUNet and original UNet present the best accuracy, while ReLayNet stays the worst in all areas, although its results have improved the most.

### 6.3. Effect of Data Augmentation

Table 6 shows the effect of applying data augmentation with each of the employed CNN methods. Since the graph search-based approach is not a machine learning method, data augmentation is not applicable here.

**Figure 7:**
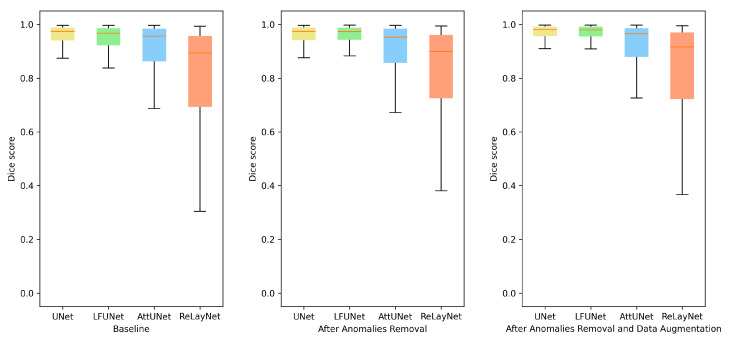
Box plots for Dice score of preretinal space for various neural network architectures.

As can be expected, the addition of more varying images helps to train the network. This strategy boosts the segmentation outcome in all the methods. A detailed analysis revealed that rotation and random cropping are the two strategies that best improve the segmentation results. This supports the observation that the angle setting for each patient is an individual parameter that can change even between examinations. Figure 7 illustrates preretinal space Dice score distribution for each of the above-discussed improvements. From the box plots, it can be deduced that not only the average value has increased, but the overall performance was also improved.

We performed a statistical test of significant differences among models (UNet, LFUNet, Attention UNet, and ReLayNet). The data used for the test is the same as those used to calculate the average values of Dice scores for Preretinal Space in Table 5. Based on the ANOVA test, the *f*-ratio value is 40.91. The *p*-value is <0.00001. The result is significant at p<0.05.

**Figure 8:**
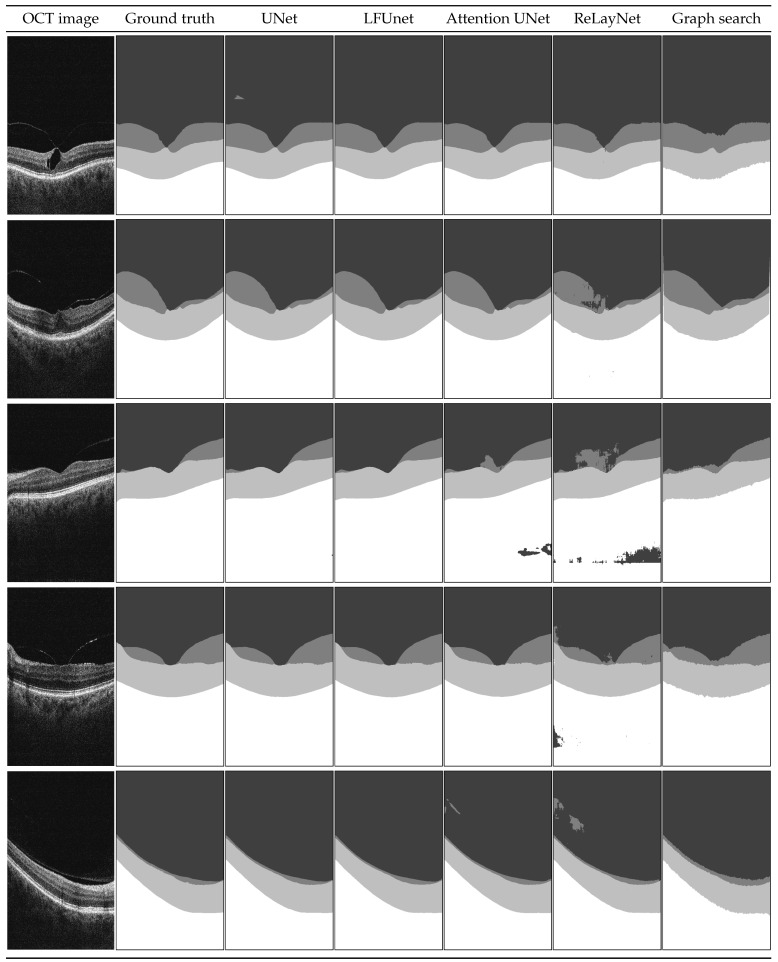
Example B-scans with corresponding reference mask and segmentation results for four analyzed neural networks and graph search-based method. Each shade in the segmentation mask represents a separate class.

Standard UNet architecture and LFUNet provide the best probability maps, although UNet has slightly better performance in segmenting preretinal space, retinal area, and PCV border. The Attention UNet and ReLayNet performed poorly here, even if their scores are better when employing data augmentation. Figure 8 presents examples of the obtained segmentation masks.

Examples shown in Figure 8 include a set of representative cases of VMT and VMA and illustrate a qualitative comparison of the obtained results. It includes two VMT cases (rows 1 and 2), two VMA cases with perifoveal vitreous detachment (examples 3 and 4), and one VMA case of slight detachment over a wide area (last row).

In the presented images, it is visible that poor evaluation scores in Table 6 for ReLayNet and Attention UNet are the effect of the network’s difficulty in discerning the areas with similar intensities (namely: vitreous, preretinal space, and the region below the retina). As an effect, patches of those classes appear in incorrect places in the prediction masks. Such occurrences are less common with UNet and LFUNet; nevertheless, those architectures are not immune to them, and further improvements are necessary.

Both UNet and LFUNet correctly learn the preretinal and retinal space borders regardless of the PCV intensity in the image, which is a significant improvement over the graph search-based method. Visually, those networks perform very well for both VMT and VMA cases. Furthermore, their accuracy is not affected by the placement of preretinal space in the image or the area it spans.

Compared with the classical approach based on image intensity gradient, the neural network learns smooth region borders and is not affected by slight local intensity variations—it can robustly generalize the preretinal space structure. Moreover, the graph search-based approach has difficulty correctly detecting the PCV border whenever it connects with the ILM line. This is a distinct disadvantage compared to the neural network methods that do not present such hindering.

On the other hand, it should be noted that in cases when the preretinal space takes only a narrow area of the image (example in the last row in Figure 8), a slight thickening of preretinal space in the prediction mask (e.g., region border 1 px higher) would significantly affect the Dice score (e.g., decreasing it by half). Such lowering of metric evaluation value may lead to the assumption that the designed network is not performing well. Nevertheless, in such a case, the MAE value would be relatively small. This is why the Dice score for regions spanning various area sizes (and particularly for imbalanced classes of small regions) should not be a sole indicator of the network’s performance.

### 6.4. Issues with Class Topology

#### 6.4.1. Preserving Layers Topology with Distance Map

To tackle the problem of topology incorrectness (in the form of another class patches in the prediction masks, as visible in Figure 8), we tested the influence of providing topology information to the network in the form of a relative distance map. Table 7 includes the results of Dice and MAE scores for four tested network architectures with each of the proposed maps and without one. Table 7 also includes the Topology Incorrectness Index to indicate how each of the methods influence network ability to discern similar classes.

When analyzing the results from Table 7, it can be noticed that for CNNs that previously performed relatively better with respect to vertical layers order (i.e., UNet and LFUNet), all maps have improved the topology, while the “CumSum” map gave the best performance with respect to Dice and MAE scores. For the other two networks (Attention UNet and ReLayNet), the “2NetPR” gives the best segmentation accuracy.

The proposed maps improve the layers’ topology from three to five times over, and both of the proposed relative distance maps (“CumSum” and “2NetPR”) perform better than the state-of-the-art approach of “2NetR”. Furthermore, the “CumSum” does not require an initial segmentation and is less computationally expensive.

Additionally, we observed that a simple linear map (“BasicOrient”) not only does not preserve correct layers topology but, in most cases, hiders network ability to segment the OCT image properly. On the other hand, for UNet, and LFUNet, this map lowered the number of images with incorrect topology.

#### 6.4.2. Improving Segmentation with Non-Typical Convolution Kernel

Previously described experiments showed the best performance of segmenting preretinal space and PCV line when utilizing UNet architecture. Therefore, this network will be used for further improvement with various convolutional kernel sizes.

**Figure 9:**
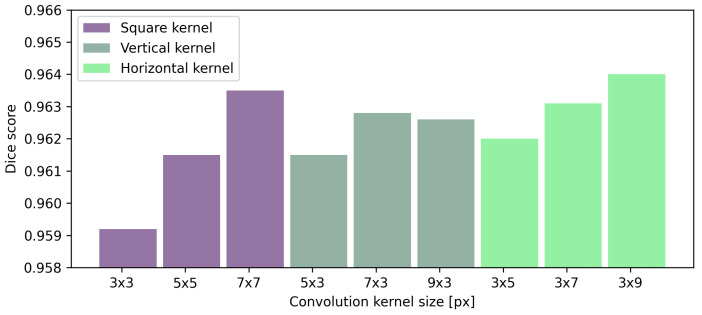
Results of preretinal space segmentation calculated using a Dice score for layer segmentation performed with UNet various kernel sizes.

Table 8 presents the effect of convolutional kernel size on the performance of preretinal space segmentation. Understandably, the average Dice score increases for every segmented region as the kernel size increases. The same observation can be made for the average MAE for all searched borders. Square kernels provide the best performance in terms of both MAE and Dice scores for the retina borders. Interestingly, the best result of preretinal space area and borders are obtained with a horizontal kernel of the size 3×9 px. From the numerical data and averaged results of Dice scores presented in Figure 9 it can be concluded that rectangular kernels (regardless of their orientation) give better results than the square ones when segmenting preretinal space. Additionally, even a kernel of size 3×5 or 5×3 px performs better than a square kernel of 5×5 px (which spans a greater area).

## 7. Conclusions

In this work, we have evaluated a set of methods employed for the segmentation of the preretinal space in optical coherence tomography images of the retina. We proposed an OCT image segmentation system that can help doctors automatically quantify morphological parameters of the preretinal space in healthy eyes and pathological cases of vitreomacular traction. Our approach provides robust end-to-end learning of preretinal space borders, with performance higher than in previous works. Employing CNN for this task does not require image preprocessing in the form of denoising, thresholding or other methods as is with standard computer vision algorithms.

We have shown the challenges associated with the segmentation of the preretinal space and proposed a solution in applying convolutional neural networks to this task. With quantitative and qualitative tests, we analyzed four neural network architectures, including UNet, LFUNet, Attention UNet, and ReLayNet for this task. Two standard metrics of Dice score and MAE were used for evaluation. An additional discussion on their interchangeability and limitation for preretinal space analysis was shown. The evaluation tests were conducted on a unique dataset of OCT images with 25 vitreomacular traction patients and 25 vitreomacular adhesion subjects. By performing the segmentation at a 2D level, thus utilizing 7050 images, we avoid computationally expensive 3D convolutions.

In general, the original UNet and LFUNet have performed relatively well, correctly segmenting preretinal space borders, with Mean Absolute Error of 1.33 px and 1.5 px, respectively, whereas the Attention UNet and ReLayNet gave MAE results of 4.02 px and 12.63 px, respectively. Nevertheless, all networks faced the challenge of incorrect vertical topology associated with the semantic image segmentation, but unacceptable in biological image analysis.

We have proposed two new approaches for improving the topological correctness of OCT image segmentation, namely two relative distance maps and the use of a non-typical convolution kernel. Extensive experiments for all network architectures show that both of the proposed relative distance maps tailored for preretinal space better preserve the correct layers’ topology (improvement from 15.1% to 3.7% for UNet, and from 11.5% to 4.8% for LFUNet), than the state-of-the-art approach (9.4% for UNet and 6.3% for LFUNet). Additionally, we conclusively demonstrate that using a bigger kernel for a UNet-type network allows improving topological correctness of segmentation to a greater extent than utilizing an additional distance map (improvement to only 2.4% of images with incorrect topology).

The presented results confirm that CNN can reliably segment preretinal space and with significantly better performance than the graph-based approach. The conducted experiments show that the best-obtained Dice score of preretinal space segmentation is up to 0.964 when using a 3×9 px kernel in a UNet architecture with 32 initial features.

## Figures and Tables

**Table 1:** Summary of neural networks used for pixel-wise semantic segmentation of retina layers in OCT images.

Network	Task	Dataset	Loss Function	Data Augmentation
ReLayNet [35]	7 layers and fluid	Duke SD-OCT public DME dataset [37] 11 B-scans each (512 × 740 px), 110 images in total	Weighted Dice and Cross Entropy Loss	horizontal flip, spatial translation, cropping
3D ReLayNet [38]	7 layers	13 volumes (13 normal subjects), 10 B-scans each, 130 images in total	Cross Entropy Loss	none
FCN8 [39]	4 layers	10 volumes (5 patients with CSC, 5 normal eyes), 128 B-scans each (512 × 1024 px), 1280 images in total	Weighted Cross Entropy Loss	none
LFUNet [40]	5 layers and fluid	58 volumes (25 diabetic patients, 33 healthy subjects), 245 B-scans each (245 × 245 px), 14210 images in total	Weighted Dice and Cross Entropy Loss	horizontal flip, rotation, scaling
DRUNet [41]	6 regions	100 scans (40 healthy, 41 POAG, 19 PACG), single B-scan through ONH each (468 px width), 100 images in total	Jaccard Loss	horizontal flip, rotation, intensity shifts, white noise, speckle noise, elastic deformation, occluding patches
Uncertainty UNet (U2-Net) [42]	photoreceptor layer	50 volumes (50 patients: 16 DME, 24 RVO, 10 AMD+CNV), 49 B-scans each (512 × 496 px), 2450 images in total	Cross Entropy Loss	none
UNet with pretrained ResNet weights [43]	4 layers	23 volumes (23 AMD patients), 128 B-scans each (1024 × 512 px), 1270 images in total	Weighted Log Loss	horizontal flip, rotation
2 cascaded UNets with residual blocks [44]	8 layers and pseudocysts	35 volumes (35 patients: 21 with macula sclerosis, 14 healthy), 49 B-scans each (496 × 1024 px), 1715 images in total	1st: Dice Loss, 2nd: MSE Loss	horizontal flip, vertical scaling

**Table 2:** Weights associated with each of the segmented class in the CAVRI dataset.

0: Vitreous	1: Preretinal Space	2: Retina	3: Region below Retina
0.1	0.5	0.29	0.11

**Table 3:** Number of images used for the training and evaluation.

Dataset	Training	Validation	Testing
All images	5608	721	721
All images −3% anomalies	5434	701	703
All images −3% anomalies + 4× data augm.	27,170	701	703

**Table 4:** The baseline Dice score and Mean Absolute Error (with Standard Deviation) results of 4 class pixel segmentation with various neural network models. The best results are highlighted in bold.

Model	Average Dice Score (SD)				MAE (SD) [px]		
	Vitreous	Preretinal Space	Retina	Region b. Retina	PCV	ILM	RPE
Graph search	0.9842 (0.033)	0.8216 (0.207)	0.9385 (0.163)	0.9888 (0.028)	7.29 (13.7)	5.10 (12.1)	5.20 (15.5)
UNet	**0.9942 (0.015)**	**0.9458 (0.088)**	**0.9929 (0.002)**	**0.9976 (0.003)**	**2.83 (6.05)**	**0.56 (0.14)**	**0.75 (0.35)**
LFUNet	0.9928 (0.017)	0.9337 (0.099)	0.9922 (0.003)	0.9972 (0.003)	3.11 (5.72)	0.60 (0.18)	0.84 (0.46)
Attention UNet	0.9822 (0.037)	0.8679 (0.197)	0.9918 (0.012)	0.9953 (0.010)	5.40 (10.04)	0.63 (0.77)	0.87 (1.11)
ReLayNet	0.9529 (0.056)	0.7827 (0.245)	0.9906 (0.008)	0.9814 (0.032)	25.66 (26.34)	0.84 (1.30)	1.45 (1.77)

**Table 5:** Dice score and Mean Absolute Error (with Standard Deviation) results of 4 class pixel segmentation with various neural network models after removing 3% of anomalous data samples. The best results are highlighted in bold.

Model	Average Dice Score (SD)				MAE (SD) [px]		
	Vitreous	Preretinal Space	Retina	Region b. Retina	PCV	ILM	RPE
Graph search	0.9853 (0.028)	0.8217 (0.206)	0.9408 (0.158)	0.9900 (0.022)	6.76 (11.1)	4.66 (9.9)	4.71 (13.2)
UNet	0.9954 (0.010)	0.9486 (0.084)	**0.9931 (0.002)**	0.9978 (0.002)	2.19 (4.55)	**0.54 (0.17)**	**0.73 (0.38)**
LFUNet	**0.9958 (0.010)**	**0.9499 (0.081)**	0.9930 (0.002)	**0.9980 (0.002)**	**2.05 (4.41)**	0.56 (0.26)	0.76 (0.52)
Attention UNet	0.9842 (0.029)	0.8688 (0.197)	0.9924 (0.004)	0.9959 (0.008)	4.89 (7.89)	0.62 (0.29)	0.82 (0.72)
ReLayNet	0.9563 (0.056)	0.7966 (0.232)	0.9908 (0.005)	0.9838 (0.026)	15.94 (18.80)	0.76 (0.79)	1.17 (1.11)

**Table 6:** Dice score and Mean Absolute Error (with Standard Deviation) results of 4 class pixel segmentation with various neural network models after removing 3% of anomalous data samples and expanding data set with 4 augmentation techniques. The best results are highlighted in bold.

Model	Average Dice Score (SD)				MAE (SD) [px]		
	Vitreous	Preretinal Space	Retina	Region b. Retina	PCV	ILM	RPE
UNet	0.9968 (0.008)	**0.9591 (0.074)**	**0.9944 (0.002)**	0.9984 (0.002)	**1.33 (2.88)**	0.50 (0.28)	0.57 (0.29)
LFUNet	**0.9973 (0.006)**	0.9590 (0.072)	0.9942 (0.002)	**0.9985 (0.001)**	1.50 (3.53)	**0.50 (0.12)**	**0.57 (0.24)**
Attention UNet	0.9850 (0.030)	0.8802 (0.186)	0.9926 (0.004)	0.9956 (0.010)	4.02 (6.54)	0.60 (0.26)	0.80 (0.73)
ReLayNet	0.9603 (0.054)	0.8015 (0.240)	0.9918 (0.004)	0.9859 (0.025)	12.63 (13.84)	0.71 (0.59)	0.96 (0.88)

**Table 7:** Dice score and Mean Absolute Error results of 4 class pixel segmentation with various distance maps for models with 3×3 px kernel size and 32 initial features after removing anomalous data samples and expanding data set with 4 augmentation techniques.

Network	Distance	Dice Score				MAE [px]			TII [%]
	Map	Vitreous	Preretinal Space	Retina	Region b. Retina	PCV	ILM	RPE	
UNet	—	0.9968	0.9591	0.9944	0.9984	1.33	0.50	0.57	15.1
	BasicOrient	0.9976	0.9606	0.9944	**0.9987**	1.42	**0.47**	0.56	4.8
	CumSum	**0.9977**	**0.9609**	**0.9945**	**0.9987**	1.25	**0.47**	**0.55**	6.8
	2NetR	0.9976	0.9601	0.9943	0.9986	1.48	0.48	0.59	9.4
	2NetPR	0.9975	0.9588	0.9943	**0.9987**	**1.20**	0.50	0.57	**3.7**
LFUNet	—	0.9973	0.9590	0.9942	0.9985	1.50	0.50	0.57	11.5
	BasicOrient	0.9975	0.9583	0.9942	**0.9987**	1.58	0.49	0.58	8.8
	CumSum	**0.9977**	**0.9605**	**0.9944**	**0.9987**	**1.22**	**0.47**	**0.56**	**4.8**
	2NetR	0.9974	0.9581	**0.9944**	**0.9987**	1.64	0.48	**0.56**	6.3
	2NetPR	0.9973	0.9571	0.9943	**0.9987**	1.26	0.51	**0.56**	5.0
AttUNet	—	0.9850	0.8802	**0.9926**	0.9956	4.02	0.60	0.80	50.5
	BasicOrient	0.9824	0.8551	0.9880	0.9962	7.31	0.87	2.05	52.9
	CumSum	0.9886	0.9025	**0.9926**	**0.9981**	4.50	**0.59**	**0.79**	38.7
	2NetR	0.9860	0.8675	0.9920	0.9980	4.74	0.72	0.98	44.1
	2NetPR	**0.9963**	**0.9508**	**0.9926**	**0.9981**	**2.04**	0.65	0.86	**12.8**
ReLayNet	—	0.9603	0.8015	0.9918	0.9859	12.63	0.71	0.96	88.3
	BasicOrient	0.9614	0.7528	0.9771	0.9930	20.00	0.59	0.79	92.0
	CumSum	0.9730	0.7937	0.9906	0.9976	15.06	0.79	1.00	79.5
	2NetR	0.9651	0.7594	0.9905	0.9974	18.55	0.70	1.11	88.1
	2NetPR	**0.9968**	**0.9544**	**0.9927**	**0.9981**	**1.42**	**0.58**	**0.81**	**10.8**

**Table 8:** Dice score and Mean Absolute Error results of 4 class pixel segmentation with various kernel sizes for UNet models after removing anomalous data samples and expanding data set with 4 augmentation techniques.

Kernel [px]		Dice Score				MAE [px]			TII [%]
Size	Area	Vitreous	Preretinal Space	Retina	Space under Retina	PCV	ILM	RPE	
3 × 3	9	0.9968	0.9591	0.9944	0.9984	1.33	0.50	0.57	15.1
5 × 5	25	0.9980	0.9615	0.9945	**0.9988**	1.08	0.47	0.54	4.6
7 × 7	49	**0.9981**	0.9635	**0.9948**	**0.9988**	0.92	**0.45**	**0.52**	4.6
5 × 3	15	0.9978	0.9615	0.9944	0.9987	1.17	0.48	0.58	5.7
7 × 3	21	0.9981	0.9628	0.9946	0.9988	0.95	0.47	0.54	2.8
9 × 3	27	**0.9981**	0.9626	0.9947	**0.9988**	0.93	0.47	**0.52**	2.4
3 × 5	15	0.9977	0.9620	0.9945	0.9987	1.22	0.47	0.55	9.7
3 × 7	21	0.9980	0.9631	0.9946	0.9987	0.98	0.46	0.55	6.8
3 × 9	27	**0.9981**	**0.9640**	0.9946	0.9987	**0.90**	0.46	0.54	5.1

## Data Availability

The OCT images data used within this study can be found at http://dsp.org.pl/CAVRI_Database/191/, (accessed on 5 November 2021), while the Python implementation code is available at https://github.com/krzyk87/pcv_segmentation, (accessed on 5 November 2021).

## Abbreviations

The following abbreviations are used in this manuscript:

AMD+CNV	Age-related Macular Degeneration with Choroidal NeoVascularization
CAVRI	Computer Analysis of VitreoRetinal Interface
CSC	Central Serous Chorioretinopathy
DME	Diabetic Macular Edeama
ILM	Inner Limiting Membrane
ONH	Optic Nerve Head
POAG	Primary Open Angle Glaucoma
PACG	Primary Angle Closure Glaucoma
PCV	Posterior Cortical Vitreous
RPE	Retina Pigment Epithelium
RVO	Retinal Vein Occlusion
VMA	Vitreomacular Adhesion
VMT	Vitreomacular Traction
